# Parental perception of child vulnerability and parental competence: The role of postnatal depression and parental stress in fathers and mothers

**DOI:** 10.1371/journal.pone.0202894

**Published:** 2018-08-27

**Authors:** Leire Gordo, Antonio Oliver-Roig, Ana Martínez-Pampliega, Leire Iriarte Elejalde, Manuel Fernández-Alcantara, Miguel Richart-Martínez

**Affiliations:** 1 Department of Social and Developmental Psychology, University of Deusto, Bilbao, Spain; 2 Department of Nursing, University of Alicante, Alicante, Spain; 3 Department of Health Psychology, University of Alicante, Alicante, Spain; Jinling Clinical Medical College of Nanjing Medical University, CHINA

## Abstract

**Introduction:**

Parents' perception that their child may be vulnerable to serious life-threatening illnesses can have negative effects on how they exercise their parenting. No studies have yet been carried out on parent´s perception of their child’s vulnerability, when the child has not suffered a severe illness. This study tries to analyze the relationship between parent´s perception of their children´s vulnerability and parental competence, and analyzes the mediating role of postnatal depression and parental stress.

**Method:**

The study was carried out on mothers and fathers of full-term infants who did not have any serious illnesses. A total of 965 people (385 fathers and 580 mothers) participated in the study.

**Results:**

The results revealed an association between parental perception of their child’s vulnerability and parent’s perception of parental competence through depression and parental stress. However, this association was different for fathers and mothers.

**Conclusion:**

The variable of perception of child’s vulnerability was a relevant factor to understand parental competence.

## Introduction

Parents' belief that their child may be vulnerable to serious life-threatening illnesses can have negative effects on both parenting and child development [1]. Green & Solnit [2] denominated these parental beliefs "Vulnerable Child Syndrome". These authors observed that in families where children suffered serious illnesses and recovered, parents and, above all, mothers, continued to be anxious about the health of their children, which exacerbated the perception that their child was vulnerable.

This parental anxiety associated with the perception that their children are vulnerable can lead to an increase in difficulties in parent-child interaction and indirectly lead to an increase in difficulties in children, such as sleeping problems or separation anxiety, difficulties in social competence and an increase of both internalizing and externalizing problems [1–5].

Several studies carried out with parents of babies who had recovered from a disease have pointed out the existence of variables linked to parental psychological distress that may be related to perception of child vulnerability. These variables include postnatal depression and parental stress [1,6–8]. However, previous studies have analyzed the perception of child vulnerability only in mothers and fathers of children who previously suffered from a serious illness. This means, as Thomasgard and Metz indicated [9] that at an early age, children do not need to have had a serious illness for parents’ perception of their child’s vulnerability to increase. This subjective concern, therefore, for the health of their children could also generate greater postnatal depression and parental stress and eventually affect parent-child relationships.

A number of studies have pointed out that postnatal depression affects not only the individual parent or child but also affects the quality of the relationship between the two [10,11]. Parents with postnatal depression tend to show less affection and sensitivity to the needs of their children, which can have negative consequences on these children’s development, such as behavioral and cognitive problems, language delays, difficulties in inhibitory control, impulsivity, response to stress, insecure attachment, and difficulties in social-emotional adjustment [12–14]. In studies on gender differences, it was traditionally pointed out that mothers showed higher levels of postnatal depression [15]. However, in recent years more attention has been paid to fathers, and it has been found that fathers also experience postnatal depression since, as for mothers, transition to parenthood can be a complicated period of adaptation to the new role [16,17]. In terms of parental stress, Bloomfield and Kendall [18] analyzed the process through which parental stress may influence children. According to these authors, parental stress negatively influences parental behavior, leading parents to perceive the experience of parenting as problematic [19–23]. Furthermore, there can be additional associated impacts such as an increase in child behavioral difficulties or even the increase or appearance of psychological distress in parents [24,25].

Regarding gender differences in parental stress, existing studies suggest that both mothers and fathers experience parental stress, but they do so differently. Mothers experience stress in relation to the role they need to exercise, and, to a lesser extent, to the social changes brought about by their new role; fathers, on the other hand, experience parental stress, but the reasons are a bit different: less because of the role they must exercise, and more because they feel that becoming parents is alienating them from their social circles [26–29]. Some authors suggest that postnatal depression and parental stress are related, since depressive symptomatology can make parents perceive reality in a more stressful way [30].

To summarize, studies seem to confirm that perception of child vulnerability may be associated with postnatal depression and parental stress, and that these variables, in turn, affect parent-child relationships [1,6,11]. However, there has been no research to deepen our understanding of parent-child relationships in situations where parents are concerned about the health of their children, though their children have not previously suffered serious illnesses.

A variable that may contribute to understanding these parent-child relationships in situations of perception of child vulnerability is “perceived parental competence”, although there have been no previous studies on this question.

Perceived parental competence is defined as "parents' beliefs or expectations regarding their ability to raise their children successfully and have some control over their child's behavior" [31 p166]. The two dimensions of parental competence are parental satisfaction and parental efficacy. Parental satisfaction is defined as the emotional component and includes frustration, anxiety and motivation associated with parenting, and parental efficacy alludes to a sense of effectiveness in relation to parenting [31].

The fact that a father or mother may have a negative self-perception of their parental competence means that they will show dysfunctional parental cognitive processes in the exercise of their parenting, which will be related to patterns of upbringing and behavioral difficulties in their children [32–34]. Conversely, perception of parental efficacy will be associated with easier transition to parenthood, a lesser tendency to perceive that their children have a difficult temperament and greater satisfaction with the social support they perceive [35,36].

Therefore, it is expected that parents’ perception of child vulnerability be related to perception of parental competence, although no research has been found in the literature on the relationship between these two variables.

### The present study

The main objective of this study was to analyze the relationship between parental perception of child vulnerability and self-perception of parental competence in parents of babies aged 6 to 8 months, analyzing the mediating role of postnatal depression and parental stress. The specific hypothesis is that postnatal depression and parental stress function like chained mediators that will mediate the relationship between parental perception of child vulnerability and parental competence. It is expected that the greater the parental perception of the child’s vulnerability, the greater parental depressive symptoms will be, which will be associated with a higher level of stress, which will in turn be linked to negative perception of parental competence.

## Method

### Participants

The sample was made up of 965 people (385 fathers and 580 mothers). The average age of the mothers was 32.77 (SD = 5.11) and that of the fathers was 35.14 (SD = 5.07). In the case of both the mothers (87.1%) and the fathers (89.4%), almost all were of Spanish nationality. In terms of the level of studies, 38.5% of the mothers and 49.8% of the fathers had primary studies, while 80.8% of the mothers and 81.9% of the fathers were married or were common-law partners. 96.8% of the mothers and 98.2% of the fathers lived with their partner. Finally, regarding the labor situation the week before the postpartum discharge, 32.4% of the mothers had been off work for 3 months or more and 82.8% of the fathers were in active employment (Table 1).

**Table 1:** Demographic features of the mothers and fathers in the study.

Parameter	Mothers (*N* = 580)	Fathers (*N* = 285)
Age (years)^a^	32.77 (5.11)	35.14 (5.07)
Spanish nationality (%)	87.1	89.4
Educational level (%):		
Can read and write	0.2	0.7
Incomplete primary education	2.5	2.8
Primary education	38.5	49.8
Secondary education	20.4	19.9
Higher education (3 years)	18.8	10.7
Higher education (>3 years)	19.6	16
Civil status (%):		
Married or common-law partner	80.8	81.9
Separated/divorced	2	2.3
Single	17	15.7
Widow(er)	0.2	0.1
Living with partner		
Continuously living with partner	96.8	98.2
Living with partner a few days a month	1.6	1.8
Does not live with any partner	1.6	0
Labor situation the week before postpartum discharge (%):		
Working	25.7	82.8
Off-work for 3 months or more	32.4	1.2
Unemployed	24.1	13
Receiving a chronic illness pension	0.3	0.2
Studying	1.4	0.8
Household chores	15.9	2
Other situations	0.2	0

^a^ Mean (*SD*)

**Fig 1:**
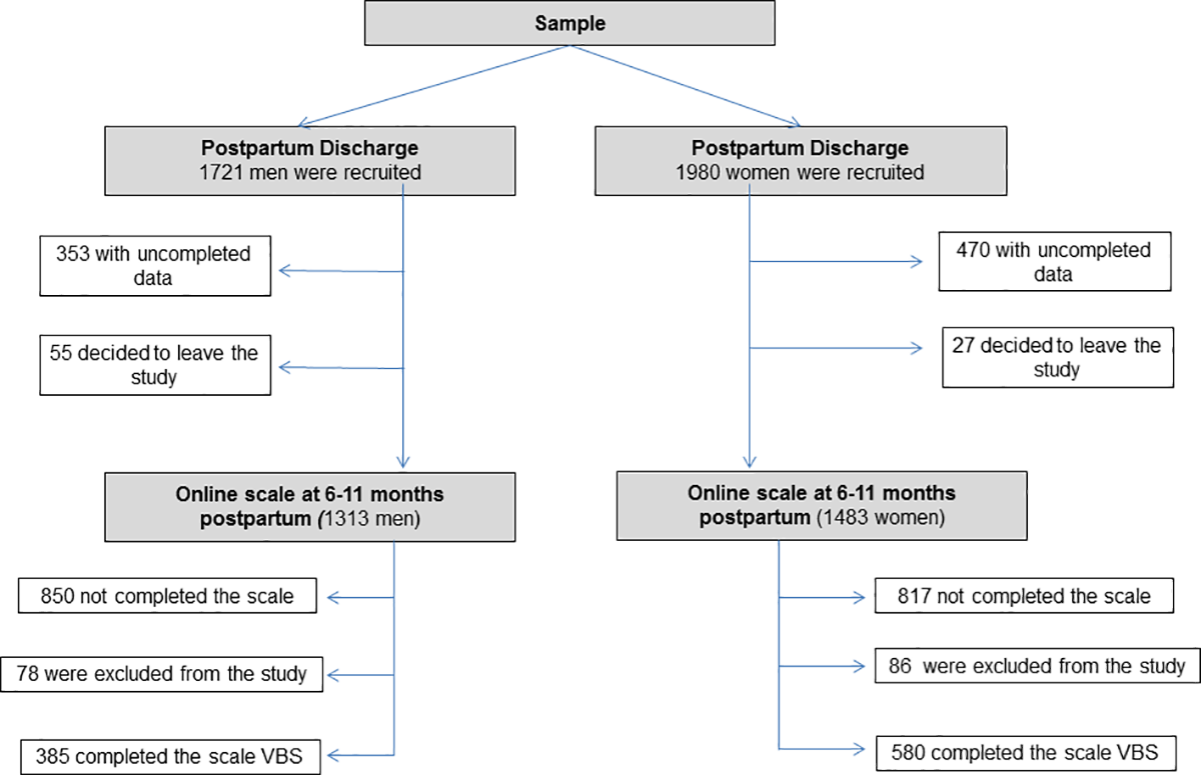
Flow diagram of study sample.

The mean age of infants was 240.13 days (SD = 42.46, range 154–445). Inclusion criteria consisted in being a mother or father of a full-term newborn delivered at a hospital, for whom pregnancy and birth was low to medium risk according to the obstetric risk classification of the Mother Care Program of the Health Department of the Valencian Community, and to speak and read Spanish without difficulty. Exclusion criteria consisted of multiple births, or severe medical maternal or newborn conditions after childbirth (Fig 1).

### Procedure

The participants in the sample were recruited on the day of postpartum hospital discharge. After obtaining written informed consent to participate in this study, the participant contact data were recorded and a battery of forms filled out. The questionnaires were the same for both mothers and fathers. Clinical data on birth and postpartum were obtained from their medical history at the moment of postpartum discharge.

Follow-up was conducted using online questionnaires. Between 6 and 11 months postpartum, participants in the study received an email with a direct link to access a self-administered form, which they could fill out using a web browser on a computer or mobile device. The link sent by email contained a unique code for each participant that allowed to interrupt the completion of the questionnaire and continue later, but prevented completing it more than once. Ten days after the email was sent, another email was sent to participants who had not sent a reply, following the same procedure as described above. A third submission was made to participants who had not responded to the questionnaire 10 days after receiving the email. Prior to sending the online questionnaire, brief reminders were sent by SMS. The study was conducted in accordance with the ethical standards of the American Psychological Association (AP A). The study was approved by the Clinical Research Ethics Committee of the General Direction of Public Health and the Higher Public Health Research Centre. This committee reports to the Valencian Health Ministry.

### Instruments

Vulnerable Baby Scale (VBS) [5]. It evaluates parental perception of infant vulnerability. It consists of 10 items *(“Sometimes I get concerned that my child does not look as healthy as she/he should”)* that must be answered on a Likert scale ranging from 1 = "*Always*" to 5 = "*I do not worry*". The internal consistency of the original scale was adequate (α = .70). There is no cut-off point defined for this scale, with higher scores indicating higher levels of child vulnerability perceived by the parents. The Spanish version showed an adequate adjustment with an RMSEA of .056 for the model and goodness of fit indexes above .90 (GFI = .97; CFI = .92) (S1 Table). The internal consistency of the Spanish version was .64 (S1 and S2 Files).

Parental Stress Scale (PSS) [20]; Spanish version [22]. This scale evaluates parental stress levels. It has 12 items structured into 2 factors: Parental Rewards (5 items) ("*I am happy in my role as a parent*") and Parental Stressors (7 items) ("*The behaviour of my child(ren) is often embarrassing or stressful to me*"). The items must be answered on a Likert scale ranging from 1 = "*Strongly Disagree*" to 5 = "*Strongly Agree*". There is no defined cut-off point, the higher the score on this scale, the higher the level of parental stress. The internal consistency of the scale was .77 for the Parental Reward factor and .76 for the Parental Stressors factor.

Edinburgh Postnatal Depression Scale (EPDS) [37]; Spanish version [38]. Parental depressive symptomatology is evaluated through this scale. It consists of 10 items ("*I have blamed myself unnecessarily when things went wrong*") that parents must answer using a Likert scale that included four levels of answers with varying meanings depending on the item. The items referred to how the parents felt in the past 7 days regarding their ability to enjoy things, optimism about the future, guilt, worry, panic attacks, coping skills, insomnia, sadness, crying and tendency to self-harm. The cut-off point defined for this scale is 10, so scores greater than 10 indicate greater depressive symptomatology. The internal consistency of the scale (α = .83) was adequate.

Parenting Sense of Competence (PSOC) [33]; Spanish version [39]. This scale assesses the feeling of parental competence through 17 items structured into two factors: Parental Efficacy (8 items) ("*If anyone can find the answer to what is troubling my child*, *I am the one*") and Parental Satisfaction (9 items) ("*Even though being a parent could be rewarding*, *I am frustrated now while my child is at his / her present age*"). The items must be answered on a Likert scale ranging from 1 = "*Strongly Disagree*" to 6 = *"Strongly Agree*". This scale does not have a defined cut-off point, the higher the score on this scale, the more positively parents perceived their parental competence. Internal consistency of the scale for the Efficacy subscale (α = .91) and Satisfaction (α = .87) was adequate.

### Strategy of analysis

In order to compare the proposed model of relationship between variables, we firstly verified the linear relationships between all variables involved in the model through Pearson correlations (*r*).

After analyzing the significance of the relations proposed in the model, the structure models corresponding to the hypothesis of the present study were defined for fathers and mothers. The Path Analysis technique and the AMOS 23 program [40], applying the maximum likelihood (ML) method, were used for its analysis. In order to evaluate the level of goodness of fit of the model, we used the Chi square test of equality of matrices (χ^2^), which should be non-significant or show low values, and the ratio between χ^2^ and the degrees of freedom of the model (χ^2^/df), which is recommended to be less than 4. However, χ^2^ is very sensitive to the size of the sample, so the Root Mean Square Error of Approximation (RMSEA) and its 90% confidence interval were used, considering values between .05 and .08 to be acceptable and values lower than .05 to be very good. Furthermore, the Goodness of Fit Index (GFI) and the Comparative Fit Index (CFI) were used, which should both show values higher than .95 [41].

To finish, we developed models based on parent gender and analyzed the significance of the difference of the chi-square values for each particular model. In the same way, we analyzed the specific differences for each of the relations established within the final model through critical ratio indexes.

## Results

As can be seen in Table 2, the scores on parental perception of their child’s vulnerability, postnatal depression, parental stress and their dimensions were below the mean in both the global sample and when the mothers and fathers were analyzed separately. However, overall scores on the perception of parental competence and its dimensions were above the mean in the total sample and were differentiated by gender. Statistically significant differences were observed as a function of the parents’ gender in postnatal depression, parental rewarding, perception of parental competence, and parental efficacy. The mothers obtained higher scores except for the dimension parental efficacy, in which the fathers obtained higher scores than the mothers. With the exception of the postnatal depression variable, there are no defined cut-off points for any of the variables; so, the clinically high and low cut-off points were calculated in the sample with reference to the percentiles 25 and 75. Regarding postnatal depression, both globally and differentiating between fathers and mothers, the averages were below the reference cut-off point for this variable. So, this population is predominantly healthy in terms of postnatal depression.

**Table 2:** Characteristics of the participants.

		N	Range	M	SD	*T*	Pc _25_	Pc _75_	As	Ku	–K-S	α Cronbach
Parental perception of their child’s vulnerability	Total	965	10–50	27.15	4.85		24	31	.11	-.29	1.96	.64
	Fathers	385		27.06	4.96	.47	23	32	.04	-.31	1.24	
	Mothers	580		27.21	4.78		24	30	.16	-2.69	1.57	
Postnatal Depression	Total	878	0–30	5.35	4.49		2	8	1.14	1.81	3.7	.88
	Fathers	342		4.54	3.89	4.48**	1	7	1.09	1.80	2.27	
	Mothers	536		5.86	4.77		2	9	1.09	1.51	2.82	
Parental Stress	Total	965	12–60	23.52	6.53		19	28	.53	.08	2.00	.83
	Fathers	385		23.39	6.43	.49	18	27	.47	-.16	1.34	
	Mothers	580		23.60	6.61		19	28	.57	.69	7.06	
Parenting Rewards	Total	965	5–25	6.30	2.08		5	7	2.81	13.41	8.35	.84
	Fathers	385		6.47	2.12	-2.13*	5	7	2.26	8.31	5.12	
	Mothers	580		6.18	2.05		5	7	3.24	17.76	7.06	
Parental Stressors	Total	965	7–35	17.22	5.35		13	21	.17	-.47	1.67	.81
	Fathers	385		16.92	5.15	1.43	13	21	.17	-.48	1.33	
	Mothers	580		17.42	5.47		13	21	.16	-.49	1.26	
Perception of parental competence	Total	965	17–102	77.77	10.84		71	85	-.44	.40	1.59	.85
	Fathers	385		76.42	10.79	3.17*	69	84	-.37	.30	.81	
	Mothers	580		78.66	10.73		72	86	-.49	.53	1.80	
Parental Efficacy	Total	965	8–48	35.23	5.78		31	39	-.30	.31	1.37	.80
	Fathers	385		33.78	5.51	6.48**	30.50	37	-.24	.34	1.34	
	Mothers	580		38.60	6.01		32	40	-.76	1.41	1.71	
Parental Satisfaction	Total	965	9–54	42.54	6.70		38	48	-.57	-.05	2.76	.80
	Fathers	385		42.64	6.83	-.38	5	7	-.60	-.13	1.92	
	Mothers	580		40.06	6.73		38	48	-.46	-.01	1.61	

Note 1: M = Mean, SD = Standard Deviation, t = Student’s t, Pc = Percentile, As = Asymmetry, Ku = Kurtosis, -K-S = Kolmogorov–Smirnov.

Note 2

*p < .05

** *p* < .001.

The distribution of parental perception of their child’s vulnerability, postnatal depression, parental stress variables and their dimensions, showed positive asymmetry, and most of the scores were located at the lower end. The Parenting Rewards dimension obtained the highest kurtosis value, which reflected data homogeneity for this variable.

Simple relations among the variables were analyzed according to the hypothesis of the present study. Parental perception of their child’s vulnerability was negatively and significantly related to perception of parental competence (r = -.12, p = .00). This association was small. If we analyze this relation according to gender, this association was also observed in mothers. However, in the case of fathers, parental perception of their child’s vulnerability was not significantly associated with perception of parental competence (Table 3).

**Table 3:** Association between parental perception of their child’s vulnerability, postnatal depression, parental stress and parenting sense of competence.

	1			2			3			4			5			6			7			8		
	T	F	M	T	F	M	T	F	M	T	F	M	T	F	M	T	F	M	T	F	M	T	F	M
1. Vulnerability	1	1	1																					
2. Depression	.19**	.23**	17**	1	1	1																		
3. Stress	.05	-.03	.09*	.47**	.42**	.50**	1	1	1															
4. Rewards	.02	-.03	.06	.36**	.38**	.38**	.68**	.71**	.66**	1	1	1												
5. Stressors	.05	-.02	.09*	.43**	.38**	.46**	.96**	.96**	.96**	.44**	.48**	.42**	1	1	1									
6. Competence	-.12**	-.05	-.17**	-.46**	-.48**	-.49**	-.65**	-.65**	-.65**	-.57**	-.60**	-.55**	-.57**	-.57**	-.58**	1	1	1						
7. Satisfaction	-.07*	-.01	-.15**	-.49**	-.46**	-.52**	-.69**	-.72**	-.66**	-.54**	-.61**	-.43**	-.63**	-.65**	-.63**	.89**	.90**	.86**	1	1	1			
8. Efficacy	-.14**	-.08	-.15**	-.29**	-.35**	-.30**	-.40**	-.39**	-.42**	-.44**	-.42**	-.49**	-.32**	-.31**	-.32**	.84**	.84**	.82**	.49**	.53**	.42**	1	1	1

Note 1

*p < .05

** *p* < .001

Note 2: T = Total, F = Fathers, M = Mothers, Vulnerability = Parental perception of child’s vulnerability, Depression = Postnatal Depression, Stress = Parental Stress, 4. Rewards = Parenting Rewards, Stressors = Parental Stressors, Competence = Parenting Sense of Competence, Satisfaction = Parental Satisfaction, Efficacy = Parental Efficacy.

Postnatal depression, both with the total sample (r = .19, p = .00) and when differentiating the father (r = .23, p = .00) and the mother (r = .17, p = .00) showed a significant and positive correlation with parental perception of their child’s vulnerability, though a small one.

Postnatal depression and parental stress showed a positive and significant correlation in both the sample total (r = .47, p = .00) and in fathers (r = .42, p = .00) and mothers (r = .50, p = .00). This association was small.

Finally, there was a positive and negative association, although a mild one, between postnatal depression and parental competence (r = -46, p = .00) and between parental stress and perception of parental competence (r = -.65, p = .00). This same relationship was also observed both for fathers and for mothers (Table 3).

Finally, the proposed model based on the hypothesis of the present study was evaluated. Table 4 shows the goodness of fit indexes of the gender-differentiated parent model. The results for the father’s model showed adequate goodness of fit in the CFI (.98) and GFI (.99) indexes. However, the RMSEA (.084) index did not reach the recommended cut-off value. Additionally, an adjustment of the data to the model was also observed in the case of mothers (GFI = .99; CFI = 99; RMSEA = .075) (Table 4).

**Table 4:** Fit indices for proposed model base on the hypothesis.

Model	χ^2^	Df	*p*	χ^2^/df	RMSEA	RMSEA IC-90%	GFI	CFI
Fathers	6.82	2	.03	3.41	.084	.021–1.57	.99	.98
Mothers	7.97	2	.02	3.98	.075	.026-.132	.99	.99

Note: χ2 = chi-square, df. = degrees of freedom, p = probability, RMSEA = Root Mean Square Error of Approximation, RMSEA (Ic-90%) = RMSEA confidence interval, GFI = Goodness of fit index, CFI = Comparative fit index.

**Fig 2:**
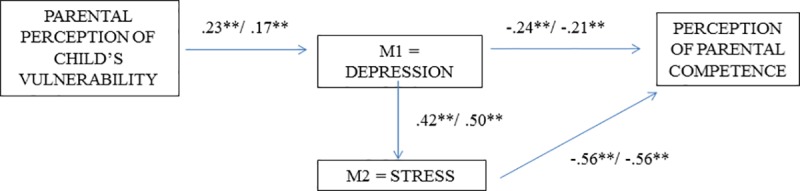
Final model of the study for fathers (left) and mothers (right).

The results presented in Fig 2 show that parental perception of their child’s vulnerability was positively associated with postnatal depression (father: β = .23, p = .00; mother: β = .17, p = .00) and that postnatal depression in turn was significantly and positively related to parental stress (father: β = .42, p = .00; mother: β = .50, p = .00). In the same way, postnatal depression (father: β = -.24, p = .00: mother: β = -.21, p = .00) and parental stress (father: paternal β = -.56, p = . 00; mother: β = -.56, p = .00) were negatively associated with perception of parental competence.

Finally, mother and father models were simultaneously evaluated based on a multigroup analysis. The chi-square difference (χ^2^) was non-significant (Δχ^2^(4) = 2,201, *p* = .70). This means that the model works in the same way regardless of parent gender.

Likewise, the critical ratio index showed an absence of differences between groups, in the specific relationships established in the model.

## Discussion

The main objective of the present study was to analyze the relationship between parental perception of child vulnerability in infants and perception of parental competence, taking into account the role that postnatal depression and parental stress exert in that relationship. Results confirmed the initial approach, since it was observed for both fathers and mothers, that when they perceived their child as most vulnerable, they also perceived themselves as less competent as fathers or mothers. This relationship has been linked to the association between parental perception of child vulnerability and increased parental depression and experienced stress. However, it is only possible to speak of real mediation in the case of the mother. In the case of the father, the relationship between parental perception of their child’s vulnerability and parental competence takes place in two steps, showing no direct relationship between parental perception of their child’s vulnerability and parental competence, and an indirect one through the association of both variables with depression and stress.

The present study articulates four relevant findings. First, the existence of an association between parental perception of child’s vulnerability and parental competence was confirmed. This result was observed for the whole sample, and was not confirmed in the case of fathers, for whom perceiving that their child could be seriously ill, did not diminish their perception of parental competence. The relationship observed between these two variables in the present study was limited, but there are no previous studies that have analyzed this relationship and with which these results can be compared. The difference in the results from the mothers and fathers may be due to gender differences in the perception of parental competence. Mothers who perceive themselves to be less competent at the parental level (less satisfaction and less self-efficacy) tend to perceive a greater vulnerability of their children to disease; this relationship is not the same in the case of the fathers. Perhaps, the results in the present study reflect the traditional roles in the distribution of family care (mothers being more focused on the care and fathers on the provision) and the emotional implications according to the gender of the parents. This is in line with the study of Moon and Hoffman [42], in which the mothers obtained higher scores than the fathers in the organization of the physical care of the children and in their emotional support.

Second, postnatal depression was associated with parental perception of child’s vulnerability, that is, parents who perceived their child most vulnerable to illness also tended to present greater depressive symptomatology. This data is in line with findings from previous research [5,43]. It should be noted that the studies that have examined these relationships were centered on the parents of babies who had recovered from an illness and not on the general population.

Third, it was observed that the existence of depressive symptoms in parents was associated with greater stress in the exercise of their parenting. That is, the greater the postnatal depression, the higher the stress levels, that is, the number of perceived stressors on the part of the parents was greater. This result also confirmed findings from other recent studies [30,44].

To finish, the study confirms the significant role of postnatal depression and parental stress in understanding lower values of parental competence with regard to parental perception of their child’s vulnerability. This is in line with other studies that found that postnatal depression causes quality of child raising to come down; that parents show less sensitivity to the needs of their children; and this in turn inevitably affects the quality of the relationship between parents and their children [11,22,45–47]. Regarding parental stress, Bloomfiel and Kendall [18] found that parents who perceived parental stress tended to be harder and more rigorous in their role as fathers or mothers, offer less support to their children, and that parent-child interaction was more conflictive.

In conclusion, it seems relevant to analyze parental perception of child’s vulnerability at the clinical level since results of this study have shown that it is associated with other variables related to parental psychological distress, which in turn affects the perception that parents have of their ability to raise their children successfully and to be able to control the behavior of their child. However, results of the present study suggest the need to continue to examine the bi-directionality of the data.

There were several limitations to this study. First, participation of fathers in the study was lower than participation of mothers. Second, the study was based on a single information source, that of fathers and mothers. Third, the internal consistency of the scale of Perception of child vulnerability is low, so future studies should continue working on its psychometric adequacy. Likewise, future studies should incorporate medical and nursing staff information. Using multi-method and longitudinal designs in data collection would also contribute to understanding causal relationships. Also, it would be interesting to increase the sample and continue the detailed analyses, studying the association between the variables considered in the present study and the parents' sociodemographic variables such as age and educational level, families in which the parents live together in the same home and families that do not, or analyzing father-mother or mother/father-baby dyads.

## Supporting information

S1 File Spanish version (VBS). (PDF)

S2 File English translation of Spanish version (VBS). (PDF)

S3 File Base Perception of child vulnerability. (SAV)

S1 Table CFA summary for the VBS. (PDF)
