# Supplementary material for: Parental perception of child vulnerability and parental competence: The role of postnatal depression and parental stress in fathers and mothers
Source: PLoS One. 2018 Aug 27;13(8):e0202894. doi: 10.1371/journal.pone.0202894 (PMC6110487; doi:10.1371/journal.pone.0202894)
Supplement: S1 File — (PDF) [file pone.0202894.s001.pdf]

“Escala de vulnerabilidad percibida del bebé”

En las siguientes preguntas, por favor marca el número que mejor describa tus pensamientos y preocupaciones sobre tu bebé en la escala del "1" al "5" que se muestra.

1. Por la noche, cuando mi bebé está durmiendo, normalmente voy a comprobar si está bien:

1) Nunca
2)
3) 1–2 veces en la noche
4)
5) Frecuentemente (al menos cada 30 minutos)

2. Si el bebé estuviera despierto y jugando, lo dejaría solo aunque estuviera a una distancia a la que no pudiera oÍrlo:

1) Nunca
2)
3) Unos 15 minutos
4)
5) Más de 1 hora

3. Si un amigo viniera de visita y tuviera un resfriado, yo:

1) No lo dejaría entrar
2)
3) Lo dejaría entrar pero no coger al bebé
4)
5) Lo dejaría entrar y coger al bebé

4. Mi bebé parece tener dolor de barriga o de otro tipo:

1) Todo el tiempo
2)
3)
4)
5) Nunca

5. Me preocupa que mi bebé no esté tan sano como debería:

1) Siempre
2)
3)
4)
5) No me preocupa

6. En general, cuando comparo la salud de mi bebé con la de otros de su misma edad, pienso que está:

1) Menos sano/a
2)
3)
4)
5) Más sano/a

Continúa en la siguiente página………

7. Me preocupa que mi bebé pueda caer gravemente enfermo:

1) Todo el tiempo
2)
3)
4)
5) Nunca

8. Me preocupa el síndrome de muerte súbita del lactante:

1) Todo el tiempo
2)
3)
4)
5) Para nada

9. Si dejara al bebé al cuidado de otra persona, ¿contactaría con ella mientras usted está fuera?

1) Si, sin duda alguna
2)
3)
4)
5) No, para nada

10. En las dos últimas semanas he contactado con un profesional de la salud por mi bebé (e.j. matrona, pediatra, médico de familia, médicos o pediatras de urgencias, enfermero de pediatría). Respecto esta última cuestión no tenga en cuenta las visitas programadas a matrona y pediatra.

1) Para nada
2)
3) Una vez a la semana más o menos
4)
5) Diariamente o más
