# Supplementary material for: Parental perception of child vulnerability and parental competence: The role of postnatal depression and parental stress in fathers and mothers
Source: PLoS One. 2018 Aug 27;13(8):e0202894. doi: 10.1371/journal.pone.0202894 (PMC6110487; doi:10.1371/journal.pone.0202894)
Supplement: S2 File — (PDF) [file pone.0202894.s002.pdf]

"Vulnerable Baby Scale " (Spanish Version)

In the following questions, please mark the number that best describes your thoughts and concerns about your baby on the scale of "1" to "5" shown.

1. At night, when my baby is sleeping, I usually go to check if (s)he's okay:

1) Never
2)
3) 1–2 Times in the night
4)
5) Frequently (at least every 30 minutes)

2. If the baby was awake and playing, I would leave him/her alone even if he/she was at a distance at which I could not hear him.

1) Never
2)
3) For about 15 minutes
4)
5) For more than 1 hour

3. If a friend came to visit and he/she had a cold:

1) I wouldn't let him/her in.
2)
3) I would let him/her in, but not hold the baby
4)
5) I would let him/her in and hold the baby

4. My baby seems to have a stomach ache or other kind of pain:

1) All the time
2)
3)
4)
5) Never

5. I am concerned that my baby is not as healthy as he/she should be:

1) Always
2)
3)
4)
5) I am not concerned

6. In general, when I compare my baby's health with that of other children of the same age, I think he/she is:

1) Less healthy
2)
3)
4)
5) Healthier

7. I am concerned that my baby may fall seriously ill:

1) All the time
2)
3)
4)
5) Never

8. I'm worried about cot death:

1) All the time
2)
3)
4)
5) Not at all.

9. If you leave the baby with another person to care for him/her, would you contact them while you were away?

1) Yes, no doubt
2)
3)
4)
5) No, not at all

10. In the last two weeks, I have contacted a health professional regarding my baby (e.g. midwife, pediatrician, family doctor, physician or emergency pediatrician, pediatric nurse). This last question does not take into account the scheduled visits of the midwife and pediatrician.

1) Not at all
2)
3) Once a week, more or less
4)
5) Daily, or more.
