# Supplementary material for: Parental perception of child vulnerability and parental competence: The role of postnatal depression and parental stress in fathers and mothers
Source: PLoS One. 2018 Aug 27;13(8):e0202894. doi: 10.1371/journal.pone.0202894 (PMC6110487; doi:10.1371/journal.pone.0202894)
Supplement: S1 Table — (PDF) [file pone.0202894.s004.pdf]

S1 Table. CFA summary for the VBS

Model	χ^2	df	*p*	χ^2/df	RMSEA	RMSEA IC-90%	GFI	CFI	Composite Reliability	A.V.E
General	127.35	32	.00	3.98	.056	.046-.066	.97	.92	.82	.41
Mothers	93.99	32	.00	2.94	.058	.044-.072	.97	.91	.72	.29
Fathers	81.91	34	.00	2.41	.061	.044-.077	.96	.91	.73	.31

Note:
χ2= chi-square, df.= degrees of freedom, p= probability, RMSEA= Root Mean Square Error of Approximation, RMSEA (Ic-90%)= RMSEA confidence interval, GFI= Goodness of fit index, CFI= Comparative fit index, A.V.E= Average Variance Extracted.
